# Dentistry in the Army

**Published:** 1899-09

**Authors:** Homer C. Croscup

**Affiliations:** Brooklyn, N. Y.


ARTICLE IX.

### DENTISTRY IN THE ARMY.

HOMER C. CROSCUP, D. D. S., BROOKLYN, N. Y.

Having had some experience in the Army during the Spanish-American war, it might be interesting to the members of the Second District Society, as well as the profession at large, to know to what extent dentistry is practiced in the service.

In the first place the Army regulations make no provision for dentists, therefore, the enlisted men, and also the officers, are put to a good deal of inconvenience and expense at times, particularly in time of war, when it becomes necessary for them to have dental attention, necessitating their being absent from the post, garrison or camp for a considerable time, especially when the nearest dentist may be located many miles away.

My connection with the Army was not in the capacity of dental surgeon, nor in the medical department, but as a line and field officer in the Fourteenth Regiment of Infantry, United States Volunteers, having served successively as First Lieutenant, Battalion Adjutant and Captain under Colonel, now Brigadier-General Frederick Dent Grant.

Thinking, however, that I would possibly be of service in my professional capacity during the campaign, I provided myself with a few forceps, lancets and other instruments for emergency cases, which I packed up with my other belongings for future use.

My regiment was mustered into the United States service at Camp Black, Long Island, on May 16, 1898, and there being a report that Spanish gun boats were dangerously near our coast, our plans for going by transport to Tampa, Florida, were abandoned, and we proceeded by rail to chickamauga, Georgia, on the following day, where we established a permanent camp, remaining there until late in August, when we were sent to Anniston, Alabama.

The nearest town to the Chickamauga camp is Chattanooga, Tennessee, eleven miles distant. At first we were fortunate in having a dentist in the hospital corps, Dr. Alfred M. Borton, who left a practice in Brooklyn to see service at the front, but who is now with the great majority, having contracted typhoid fever soon after the regiment reached the South, from which he died after a few days' illness. Truly a hard death for a soldier.

It was then that I began to realize that I was likely to be kept busy in the dual capacity of dental surgeon and company commander, there being about thirteen hundred men in my regiment, and five other regiments of different branches of the service, with about the same number of men each, stationed in close proximity. I found they were as badly off so far as dentists were concerned as the 14th; in fact much worse. As far as I have been able to learn I was the only dentist, after the death of Dr. Borton, in the entire first division of the Third Army Corps.

It became necessary for me to systematize my work in the dental line, as my other duties were various and did not permit of interruptions, so I arranged to be at the hospital tent one hour each day to attend all comers, and nearly every day a line of from twenty to thirty awaited my arrival.

Of course, extraction, in the majority of cases, was the necessary treatment under the circumstances, but if I had been prepared in the capacity of dental surgeon, nearly all the teeth thus sacrificed could have been saved.

In many cases the teeth had been broken by accident; it would have been an easy matter to extirpate the pulps and prepare the teeth for crowning. Many of these accidents occurred in the regular drills and exercises, while serving under the government and under orders, and the least Uncle Sam could do, would be to give them proper care and treatment.

I remember upon one occasion after a drill in battle formation, extended order, two men came to me suffering from fractured incisors, one having been struck in the mouth with the butt of a rifle, breaking off the right superior central and lateral at the gum margin. From both roots the pulps protruded. I prepared sharp pieces of wood dipped in carbolic acid and forced them into the canals. This is an ancient practice and it came in handily at that time. I have subsequently placed crowns on both roots.

I had many cases from other regiments where the surgeons had made heroic attempts to extract refractory molars, finally sending them to me to finish up. They, however, could hardly have expected to be successful with the instruments at their command.

Many lesions commonly observed in dental practice were met with, such as pulpitis, pericementitis, alveolar abscess, pyorrhea, epulis, diseases of the antrum, etc., etc.

Of course, only a small proportion of the Army came under my personal observation, but that was sufficient to demonstrate the fact that it is a duty which the Government owes its soldiers to make provision for the proper care of their teeth, and to provide dentists as a recognized part of the Army establishment.

Of course, the fine, delicate and intricate operations could not well be performed in the field, but good, honest and conscientious dentists could always find a way to give good and faithful service, even under the most adverse circumstances, and this would be a great benefit to both officers and men. On the other hand, many young dentists would be glad of the opportunity to serve in the Army as dental surgeons, even though the remuneration be not as lucrative as in a private practice.

When the Hull bill was formulated, it provided for one hundred dentists in the Army, with the rank and pay of First Lieutenants. That would be about $1,800 per year. Unfortunately, this bill was so defaced previous to its passage by Congress that it was hardly recognizable, and the section relating to dentists was stricken out to make room for something else. Knowing, however, the advantage this would be to the service I am still hopeful that it may yet becoms a law.—*Items of Interest.*

---

### Monthly Summary.

